# Toward a compact hybrid brain-computer interface (BCI): Performance evaluation of multi-class hybrid EEG-fNIRS BCIs with limited number of channels

**DOI:** 10.1371/journal.pone.0230491

**Published:** 2020-03-18

**Authors:** Jinuk Kwon, Jaeyoung Shin, Chang-Hwan Im

**Affiliations:** 1 Department of Biomedical Engineering, Hanyang University, Seoul, Korea; 2 Department of Electronic Engineering, Wonkwang University, Iksan, Korea; Columbia University, UNITED STATES

## Abstract

It has been demonstrated that the performance of typical unimodal brain-computer interfaces (BCIs) can be noticeably improved by combining two different BCI modalities. This so-called “hybrid BCI” technology has been studied for decades; however, hybrid BCIs that particularly combine electroencephalography (EEG) and functional near-infrared spectroscopy (fNIRS) (hereafter referred to as hBCIs) have not been widely used in practical settings. One of the main reasons why hBCI systems are so unpopular is that their hardware is generally too bulky and complex. Therefore, to make hBCIs more appealing, it is necessary to implement a lightweight and compact hBCI system with minimal performance degradation. In this study, we investigated the feasibility of implementing a compact hBCI system with significantly less EEG channels and fNIRS source-detector (SD) pairs, but that can achieve a classification accuracy high enough to be used in practical BCI applications. EEG and fNIRS data were acquired while participants performed three different mental tasks consisting of mental arithmetic, right-hand motor imagery, and an idle state. Our analysis results showed that the three mental states could be classified with a fairly high classification accuracy of 77.6 ± 12.1% using an hBCI system with only two EEG channels and two fNIRS SD pairs.

## Introduction

Brain-computer interfaces (BCIs) are an emerging technology that provides people who lost their normal pathways for communication with an alternative communication channel by decoding their neural signals [1–3]. For example, Han et al. [4] implemented an electroencephalography (EEG)-based BCI system for online binary communication with a patient in completely locked-in state, and demonstrated high online classification accuracy of 87.5% in discriminating left-hand imagery and mental subtraction tasks. The brain activity of the BCI user is captured via various neuroimaging modalities and is then translated into certain commands by which the user can communicate with the external world. The neuroimaging modalities that can be used in BCI implementations are categorized into invasive and noninvasive methods according to the requirement (or not) of invasive surgery to implant neural-signal sensors into the brain. EEG and functional near-infrared spectroscopy (fNIRS) are the two major neuroimaging modalities most frequently employed for implementing noninvasive BCIs. These methods have several advantages over invasive methods, such as being safer and offering high accessibility, a more affordable cost, scalability, and portability [5–9]. With the rapid development of BCI technology, increasing interest has been drawn toward hybrid BCIs, which are combinations of two or more BCI systems. Among the various possible hybrid BCIs, the hybrid EEG-fNIRS BCI (hereafter referred to as *hBCI*) is the most widely studied because of the complementary characteristics of EEG and fNIRS. Some recent studies, including those of Fazli et al. [6], Shin et al. [10], and Khan et al. [11, 12] demonstrated that hBCIs could achieve a better overall performance compared with unimodal BCIs in terms of classification accuracy and information transfer rate (ITR). These improvements in performance are thought to originate from the synergetic effect caused by the high temporal resolution of EEG and the relatively lower variability of fNIRS [13, 14].

Recently, BCI researchers have been gradually focusing on implementing practical BCI systems, as indicated by Hwang et al. [15]. Therefore, the application fields of BCIs have extended to various others, such as neuro-rehabilitation, neuro-feedback, assistive and humanoid robots, the gaming and entertainment industry, and smart-home services [16–19]. However, hBCI systems have rarely been used in practical applications yet because many sensors are required to capture two different types of brain signals simultaneously, thus making the overall hardware of such systems bulky and complex. Moreover, time-consuming preparatory steps, such as the placement and attachment of the signal sensors, have to be carried out before each use of the hBCI system, which consequently lowers its practicality. To circumvent this issue, we have to develop a compact hBCI system by reducing the number of signal sensors while maintaining an overall BCI performance level that is high enough for use in practical applications.

In our previous study [20], we demonstrated the feasibility of developing a three-class hBCI system that can successfully discriminate between three types of brain signals induced by mental arithmetic (MA), motor imagery (MI), and idle state (IS) tasks. This hBCI system achieved a fairly high classification accuracy of 82.2 ± 10.2% and a satisfactory ITR of 4.70 ± 1.92 bit/min; however, the large numbers of signal sensors required (21 electrodes for EEG and six source-detector (SD) pairs for fNIRS) made the preparation time prolonged and some participants felt tired even before starting the main experiments. In this study, we demonstrate the feasibility of implementing a compact ternary hBCI system with minimum numbers of EEG channels and fNIRS SD pairs. The proposed system can discriminate between three types of mental states with a classification accuracy high enough to be used in practical settings.

## Materials and methods

In this section, the experimental paradigms and analysis methods used are described in detail. Note that some of the descriptions in this section were adopted from our previous study [20].

### Dataset

**Fig 1:**
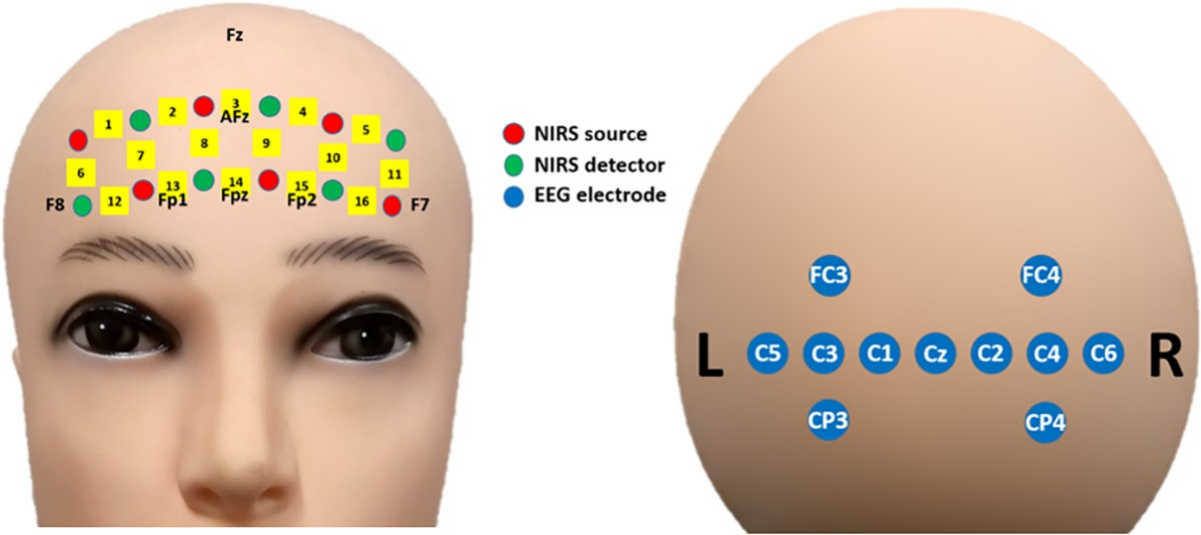
**Arrangement of EEG channels (blue) and fNIRS optodes (red: Sources, green: Detectors) on the frontal (left) and motor (right) areas.** Channels were numbered in the same manner as in a previous study [20].

We used a part of the EEG and fNIRS datasets used in our previous study [20]. The original dataset consisted of 21-channel EEG (with a bipolar vertical EOG channel) and 16-channel fNIRS data of 18 healthy adult participants (10 males and 8 females, 23.8 ± 2.5 years of age). From the original dataset, EEG data recorded at 11 central channels (without a vertical EOG channel) and fNIRS data measured at all 16 prefrontal channels were selectively used. The location of the EEG and fNIRS channels used in the present study are shown in Fig 1. Channels were numbered in the same manner as in the previous study [20].

### Experimental paradigm

**Fig 2:**
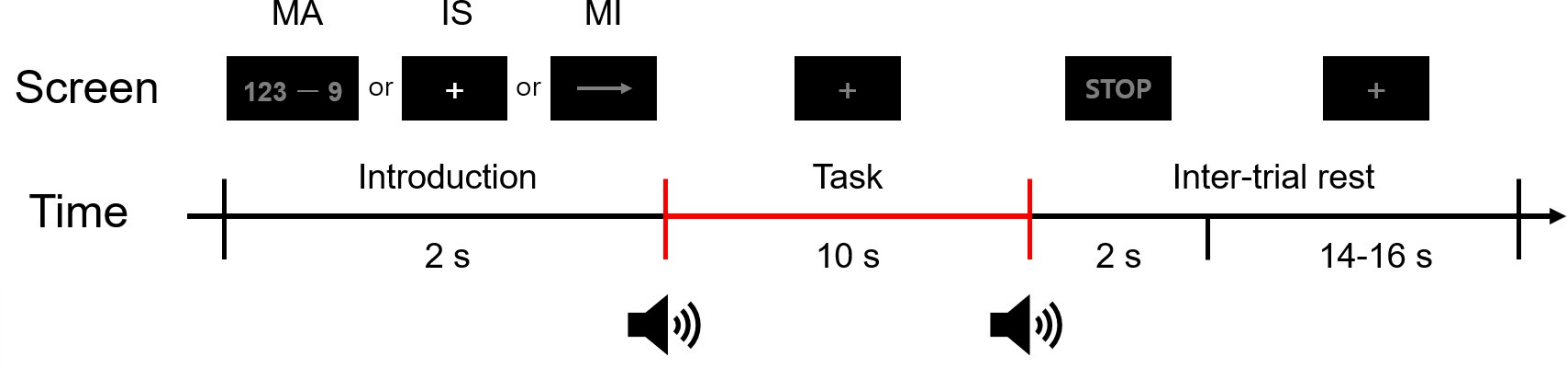
Illustration of a single trial of the experiment. Each trial consisted of an introduction period of 2 s, a task period of 10 s, and an inter-trial rest period (stop and rest) of 16–18 s. During the introduction period, a random task (MA, MI, or IS) was displayed to the participant. After a short beep, the participant performed the task displayed in the introduction period while looking at a fixation cross. When a STOP sign was displayed with a second short beep, the participants stopped performing the task and relaxed during the random-length inter-trial rest period.

Fig 2 shows the experimental paradigm used to generate the EEG and fNIRS dataset. Each trial consisted of instruction (2 s), task (10 s), and inter-trial rest (16–18 s) periods. During the instruction period, a right-hand MI, MA, or IS task was randomly selected. For the right-hand MI tasks, a right arrow was presented to the user, whereas an expression consisting of a three-digit number minus a one-digit number between 6 and 9 (e.g., 123 − 9) was randomly presented for the MA tasks. For the IS tasks, a fixation cross was displayed at the center of the monitor. During the task period, the participants were asked to perform the designated task. For the right-hand MI tasks, the participants imagined complex kinesthetic finger tapping, namely tapping their fingers, in the order of second (index finger), third (middle finger), fourth (ring finger), fifth (little finger), fourth, third, second, and first (thumb) repeatedly at a rate of approximately 2 Hz. For the MA tasks, the participants were instructed to subtract the one-digit-number from the three-digit number displayed during the instruction period and then continuously subtract the one-digit number from the result of the previous calculation as fast as possible (e.g., 123 − 9 = 114, 114 − 9 = 105, 105 − 9 = 96…). For the IS tasks, the participants had to remain relaxed without performing any specific mental imagery task. These three tasks were performed 30 times each. Before the experiment, all participants underwent a pre-training session with visual feedback to help them produce appropriate MI-related brain activation patterns. While the participants executed actual finger tapping tasks, their θ (4–8 Hz), α (8–13 Hz), and β (13–30 Hz) band powers extracted from the EEG signals were displayed on the monitor in real-time. Additionally, while the participants were performing kinesthetic motor imagery (not visual motor imagery [21]), they were instructed to try to make the band powers match as closely as possible those observed during actual finger tapping execution. This motor imagery training procedure lasted until the participants could reproduce consistent band power patterns during the motor imagery tasks, similar to those observed during the actual finger tapping tasks. The average training time was approximately 15 min. Even though some participants had difficulty in reproducing consistent task-related EEG signals, the training time was limited to 30 min to avoid the potential influence of the participants’ fatigue on the data recording. Thus, this preliminary MI training procedure lasted from 5 to 30 min depending on the participant.

### Preprocessing

MATLAB 2017a (MathWorks; Natick, MA, USA) was used to analyze EEG and fNIRS data, and some functions implemented in the EEGLAB (https://sccn.ucsd.edu/eeglab/index.php) and BBCI toolboxes (https://github.com/bbci/bbci_public) were employed [22, 23]. EEG data were first downsampled to 200 Hz and bandpass-filtered to 1–50 Hz with a 6^th^-order Butterworth zero-phase filter. As for the fNIRS data, the raw optical densities (ODs) were converted to concentration changes of deoxy/oxy-hemoglobin (ΔHbR/ΔHbO) using the following formula [24]:

$$\left(\begin{array}{c} \Delta HbR\\ \Delta HbO \end{array}\right)=\left(\begin{array}{ccc} 1.8545 & -0.2394 & -1.0947\\ -1.4887 & 0.5970 & 1.4847 \end{array}\right)\left(\begin{array}{c} \Delta OD_{780}\\ \Delta OD_{805}\\ \Delta OD_{830} \end{array}\right)\;(mM\cdot cm)$$

where ΔODs represent the optical density changes at wavelengths of 780, 805, and 830 nm. The converted ΔHbR and ΔHbO values were then bandpass-filtered to 0.01–0.09 Hz with a 6^th^-order Butterworth zero-phase filter.

### Feature extraction

The EEG data were segmented into epochs from 0 to 10 s (task period) with respect to the task onset (0 s). To apply the filter bank common spatial pattern (FBCSP) algorithm, we applied a filter bank (6^th^-order Butterworth zero-phase filters) with multiple passbands of *θ*, *α*, and *β* waves to each EEG epoch. The multiple passbands were chosen considering the participant-dependency of the task-relevant frequency band, as reported by a previous study [25]. The number of CSP components (*k*), which depends on the number of EEG channels, was determined as follows:

k=min(n,6),(2≤n≤11)

where *n* is the number of EEG channels used and min (*a*, *b*) returns the smaller value between *a* and *b*. The EEG feature vectors were constructed using either the log-variance of *k* CSP components (*n* ≤ 6) or the first and last three CSP components (*n* > 6) sorted by their typical eigenvalue score. Hence, a total of *k* × 60 EEG features were generated (*k* CSP components × [3 passbands × 20 trials]).

The fNIRS data were segmented into epochs from −1 to 15 s considering that the hemodynamic delay is in the order of several seconds [27]. The baseline of the filtered data was corrected in each channel by subtracting the temporal mean amplitudes of the data within the [−1 0] interval for each fNIRS epoch. fNIRS feature vectors were constructed using the temporal mean amplitudes for multiple temporal windows of 0–5, 5–10, and 10–15 s for each epoch. This approach using multiple windows is known to result in higher classification accuracy than using a single temporal window [20, 26]. The dimension of the fNIRS feature vectors was ([number of fNIRS channels × 2 fNIRS chromophores] × [3 periods × 20 trials]).

### Classification

**Fig 3:**
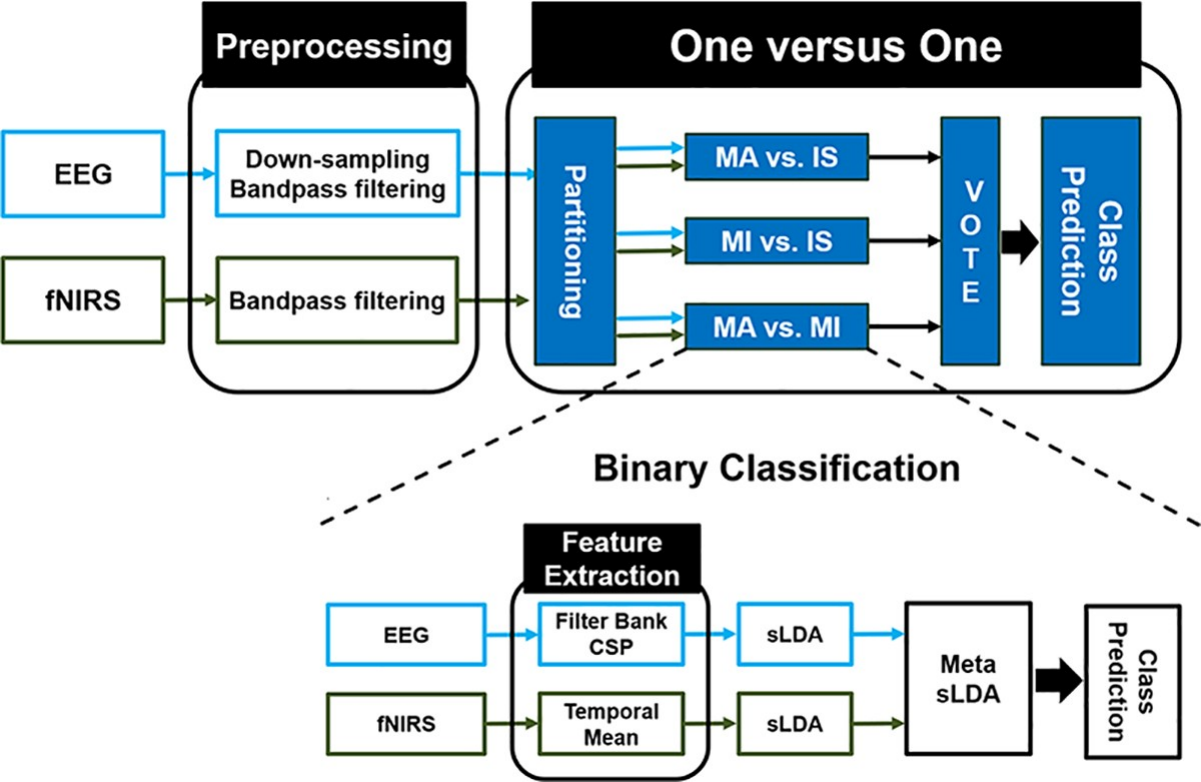
Data processing procedure. CSP and sLDA stand for common spatial pattern and shrinkage linear discriminant analysis, respectively. To perform meta-classification, we concatenated the outputs of individual EEG and fNIRS classifiers to construct feature vectors for the meta-classifier. The “one-versus-one” block represents the strategy used to solve the three-class classification problem by dividing it into three binary classification problems and employing majority voting (VOTE) based on the result of each binary classification to predict the class.

To perform three-class classification, we applied the one-versus-one (OVO) classification strategy because it is impossible to directly apply the CSP filter to multi-class classification problems. Therefore, the three-class classification problem was decomposed into three binary classification problems (i.e., MA vs. MI, MA vs. IS, and MI vs. IS). For each of the possible pairs of classes, binary classifications were performed using the shrinkage linear discriminant analysis (sLDA) method. Shrinkage is a tool to improve the estimation of covariance matrices in situations where the number of training samples is small compared to the number of features. This method is known to mitigate the loss of classification accuracy due to the use of high-dimensional feature vectors by employing a shrinkage parameter based on the Ledoit–Wolf lemma [27–30]. To properly combine the EEG and fNIRS features, we adopted a meta-classification method known to yield better classification accuracy than conventional methods based on simply concatenating both types of features. Two individual classifiers were trained using either the EEG or fNIRS feature vector sets, and then the outputs of the EEG and fNIRS classifiers were combined to construct new feature vectors for the meta-classifier [6, 10, 31]. After the three binary classification results were acquired, majority voting was used to predict the final class. Classification accuracy was evaluated via a 10 × 10-fold cross-validation. This procedure randomly divides the dataset into ten equal-sized partitions. Nine partitions are used for training the classifier and the remaining partition is used for testing the performance of the classifier. This procedure is repeated ten times and the final classification accuracy is calculated by averaging the ten separate results. Fig 3 illustrates the whole data-processing and classification procedures.

### Selection of the optimal sensor configuration

Among the central EEG channels covering the motor area [32–34], the optimal channels were chosen based on an MI vs. IS classification accuracy criterion under the assumption that a more accurate MI vs. IS classification result would also result in a more accurate three-class classification result. Likewise, the optimal fNIRS SD pairs were chosen based on an MA vs. IS classification accuracy criterion.

The optimal EEG channels were chosen via the following two steps:

(Step 1) We first determined the minimum number of EEG channels (denoted by *m*) required to achieve an average classification accuracy for MI and IS tasks surpassing the threshold of an effective BCI (70%) [35]. Starting from 11 EEG channels, the number of EEG channels was reduced until only two EEG channels remained by using the sequential backward selection (SBS) algorithm because CSP filters require EEG data from at least two channels. For a given number of EEG channels, the optimal combination of EEG electrodes that yielded the highest classification accuracy for MI and IS tasks was determined independently for each participant by repeatedly computing the classification accuracy for all possible combinations of EEG electrodes.
Step 2) After the determination of the value of *m*, a common set of *m* EEG channels (i.e., an EEG configuration with *m* electrodes) that could generally maximize the MI vs. IS classification accuracy averaged across all participants was determined.

As for the fNIRS data, the number of SD pairs is more important than the number of channels because the size of an fNIRS system mainly depends on the number of SD pairs rather than that of channels. Therefore, the optimal number of lattice-arranged SD pairs was explored based on an MA vs. IS classification accuracy criterion by decreasing the number of SD pairs from six to one.

### Statistical analysis

Statistical analyses were also performed using MATLAB 2017a. Because the normality criterion was not satisfied owing to the small sample size used, non-parametric testing was employed. A Friedman test was conducted to verify if there were significant differences among the classification accuracies attained using different sorts of sensor arrangements. A Wilcoxon signed rank test was used for post-hoc analyses, for which the *p*-values were corrected using the false discovery rates (FDRs). The p-values of less than 0.05 were considered statistically significant and all confidence intervals (CIs) were calculated at the 95% level using the following formula:

$$tanh(arctanh(r)\pm 1.96/\sqrt{n-3}),$$

where *n* is the sample size and *r* is the Spearman correlation coefficient. The effect size was calculated to investigate the magnitude of differences using the following equations [36]:

$$Effect\;Size=\frac{Z}{\sqrt{N}},$$

where Z is the test statistics of the Wilcoxon signed rank test and *N* is the total number of observations.

## Results

**Fig 4:**
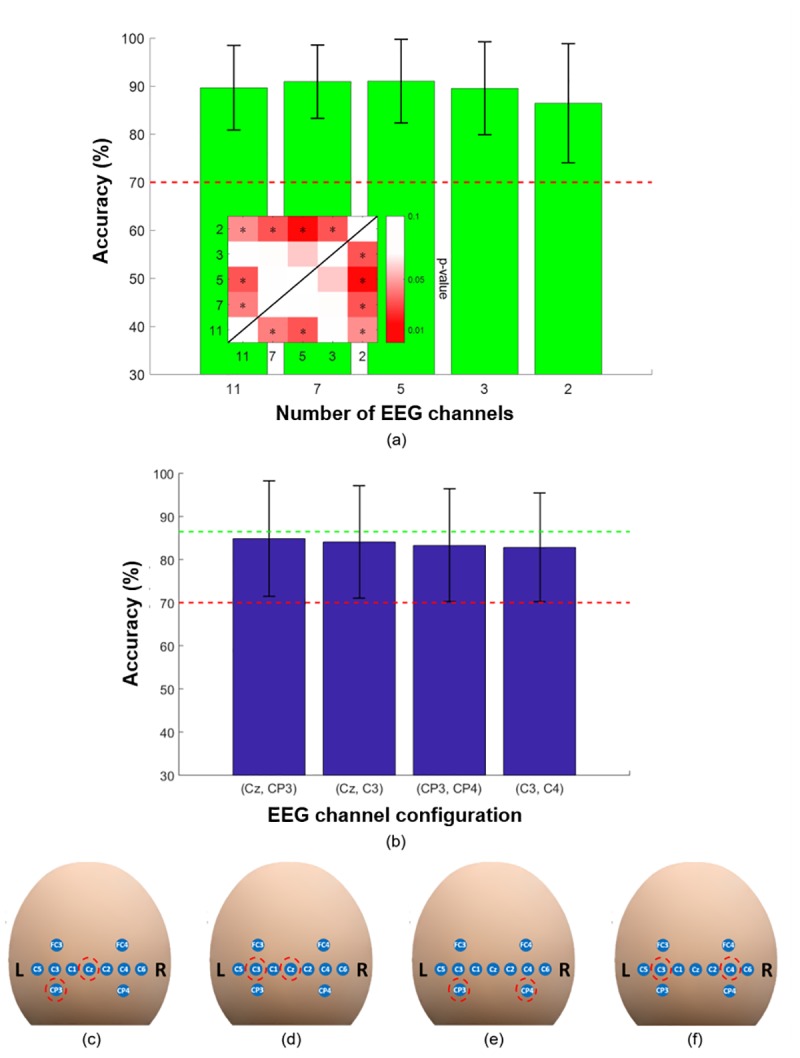
(a) MI vs. IS EEG classification accuracies as a function of the number of EEG channels. Green bars indicate the grand average classification accuracies calculated using the sequential backward selection algorithm. The color map indicates the statistical significance between the differences in classification accuracy calculated according to the number of EEG channels (*p < 0.05). (b) MI vs. IS EEG classification accuracies calculated using the four optimal configurations with two EEG channel, which were (c) (Cz, CP3; the best), (d) (Cz, C3), (e) (CP3, CP4), and (f) (C3, C4; the fourth best). The red and green horizontal dashed lines indicate the threshold for an effective BCI (70%) and the value corresponding to the two EEG channels (x-axis) shown in (a), respectively. The error bars indicate standard deviation.

Fig 4(A) shows the MI vs. IS EEG classification accuracies attained according to the number of channels; the error bars indicate standard deviation. The red horizontal dashed line represents the threshold of an effective BCI (70%). The average classification accuracies attained when using eleven, seven, five, three, and two EEG channels (green bar graphs) were 89.7 ± 8.8%, 91.0 ± 7.6%, 91.1 ± 8.7%, 89.6 ± 9.7%, and 86.5 ± 12.4% (mean ± standard deviation), respectively. The statistical significance between the different cases is shown in the inset of Fig 4(A) as a *p*-value color map table, where statistically significant pairs are denoted by asterisks. Although statistically significant differences were found in most cases, the absolute differences were not very large. Based on these results, the minimal number of EEG channels for attaining an average classification accuracy that surpasses the effective BCI threshold was found to be two. In the next step, the average classification accuracies for all 27 possible EEG channel pairs were calculated. Fig 4(B) shows the classification accuracies attained using the four EEG channel pairs indicated by red dashed-line circles in Fig 4(C)–4(F), which were the highest classification accuracies. The green horizontal dashed line indicates the classification accuracy averaged across all participants when individually optimal EEG channel pairs were used instead of a common EEG channel configuration (86.5 ± 12.4%, as indicated in Fig 4(A)). All the results are summarized in S1 Table included in Supplementary Information file. As shown in Fig 4(B), the four best channel pairs yielded classification accuracies of 84.8 ± 13.4%, 84.1 ± 13.0%, 83.3 ± 13.1%, and 82.8 ± 12.5%. No significant differences in classification accuracy were found among them. Out of the four channel pairs, the (Cz, C3) channel pair, which was the second best in terms of classification accuracy, was selected instead of the (Cz, CP3) channel pair, which was the best channel pair. This was done because we think that the (Cz, C3) channel pair is much easier to implement in a practical wearable system, as readily shown in Fig 4(D).

**Fig 5:**
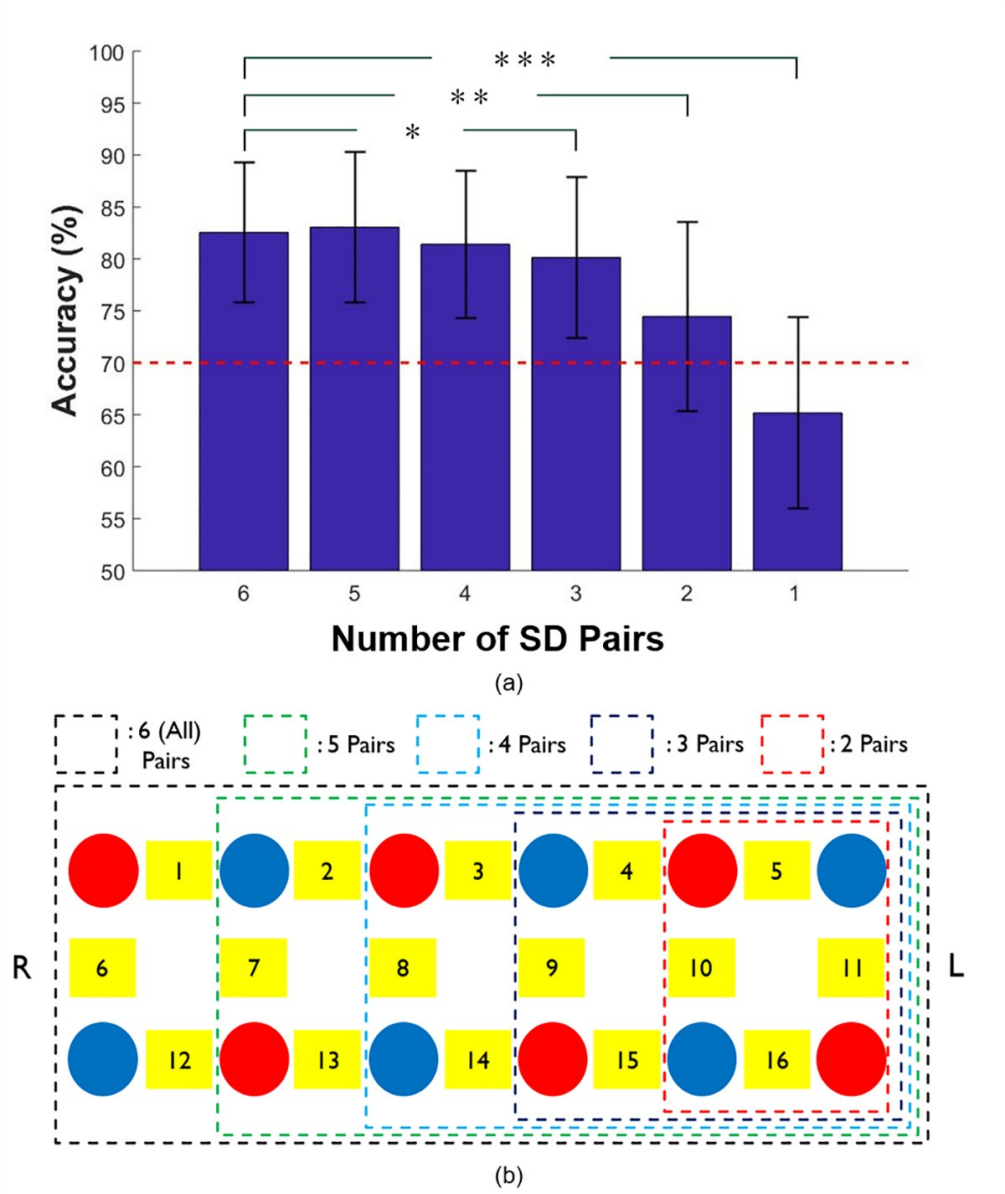
(a) Highest MA vs. IS fNIRS classification accuracies as a function of the number of SD pairs shown in (b). Statistical significance was calculated between the classification accuracies achieved using all the SD pairs and various numbers of SD pairs (*p < 0.05, **p < 0.01, ***p < 0.001). The error bars represent the standard deviation.

As for the fNIRS data, the MA vs. IS classification accuracies according to the number of SD pairs are shown in Fig 5(A), in which the error bars indicate standard deviation. In total, twenty different SD pair arrangements were tested as summarized in S2 Table in the Supplementary Information file. The highest classification accuracies achieved with six, five, four, three, two, and one SD pairs were 82.6 ± 6.7%, 83.1 ± 7.3%, 81.4 ± 7.1%, 80.1 ± 7.7%, 74.4 ± 9.5%, and 65.2 ± 9.2%, respectively (see Fig 5(B) for the optimal SD pair arrangements for different numbers of SD pairs). Compared with the classification accuracy achieved when using all SD pairs, a statistically significant loss of classification accuracy was observed when the number of SD pairs was less than four. In addition, we found that the classification accuracy for a single SD pair case dropped under the effective BCI threshold, implying that at least two SD pairs should be employed to implement a practically feasible BCI. Therefore, we expected that two SD pairs representing the left dorsolateral prefrontal cortex (left DLPFC) (Ch 5, 10, 11, and 16) or the right DLPFC (Ch 1, 6, 7, and 12) would be the best choices considering the important role of the DLPFC in cognitive task performance. The arrangements of SD pairs that yielded the highest MA vs. IS classification accuracy for each number of SD pairs are shown in Fig 5(B). The optimal SD pairs were mostly located in the left prefrontal area, which is in line with the important role of the dorsolateral prefrontal cortex in cognitive task performance and previous studies reporting that brain activity in the left prefrontal area is closely associated with mental arithmetic tasks [37]. More detailed results are summarized in Table 2 in S1 Text.

Table 1 shows the classification accuracies of three-class hBCIs that included the (Cz, C3) EEG channels but had different numbers of fNIRS SD pairs. The average hBCI classification accuracies were 77.0 ± 9.3%, 78.2 ± 9.7%, 78.9 ± 9.9%, 78.8 ± 10.6%, 77.6 ± 12.1% when the six, five, four, three, and two SD pairs that are depicted in Fig 5(B) were employed, respectively. No significant differences in classification accuracy were observed among the systems. We then compared the three-class classification accuracies of the hBCIs with those of an EEG-based BCI that solely used the data from (Cz, C3) EEG channels, for which the results are listed in the rightmost column of Table 1. The average EEG classification accuracy was reported to be 72.6 ± 13.9%, demonstrating the advantage of employing hBCIs in ternary BCI applications. Statistical tests showed that the classification accuracies of the EEG-only BCI and the hBCIs differed significantly when the number of SD pairs was two, three, and four (corrected *p* < 0.05). Since we selected the (Cz, C3) EEG channels instead of (Cz, CP3), which showed the highest classification accuracy, we also calculated the average classification accuracies for three-class hBCI when the (Cz, CP3) EEG channels were selected. The results were 77.9 ± 10.1%, 78.5 ± 10.1%, 79.1 ± 10.8%, 78.9 ± 10.7%, 76.4 ± 10.5% when (Cz, CP3) EEG channels were used with six, five, four, three, and two SD pairs, respectively. The results indicate that there were no big differences between the classification accuracies achieved using (Cz, CP3) and (Cz, C3). Our results show that the three mental states could be classified with a high classification accuracy of 77.6 ± 12.1% using an hBCI with only two EEG channels and two fNIRS SD pairs.

**Table 1:** Individual three-class hBCI classification accuracies.

fNIRSParticipant	All	5 Pairs	4 Pairs	3 Pairs	2 Pairs	EEG only(Cz, C3)
1	74.8	79.8	80.8	80.1	82.6	79.1
2	81.0	82.3	80.6	82.7	81.7	79.3
3	74.7	76.8	77.2	78.7	77.3	79.3
4	89.9	92.4	92.7	93.6	91.1	86.3
5	66.0	63.3	59.4	60.2	58.3	55.8
6	90.4	93.2	94.6	94.1	94.8	88.7
7	62.2	71.3	74.3	72.1	72.0	72.4
8	71.2	73.6	75.2	72.9	74.1	70.1
9	79.1	83.1	84.1	86.9	87.7	75.6
10	78.8	80.4	81.4	82.0	84.4	80.1
11	87.3	90.4	91.0	92.4	91.9	91.3
12	74.8	72.8	73.8	75.7	68.1	63.2
13	57.6	55.9	58.0	57.3	55.9	58.7
14	78.8	77.7	78.4	70.3	62.7	50.0
15	70.8	73.4	74.9	72.9	67.7	54.9
16	84.7	78.1	76.4	75.2	71.0	52.3
17	87.8	89.3	90.2	91.0	94.3	94.8
18	76.1	74.0	77.8	81.0	80.6	74.6
**Mean**	**77.0**	**78.2**	78.9*	78.8*	77.6*	**72.6**
**Std**	**9.3**	**9.7**	**9.9**	**10.6**	**12.1**	**13.9**

Individual three-class hBCI classification accuracies attained using the optimal EEG channel pair (Cz, C3) and different numbers of SD pairs. The three-class EEG classification accuracy results for the (Cz, C3) channel pair are shown in the right-end column. The statistical significance of the differences between the classification accuracies calculated using EEG channels only (right-end column) and both EEG and fNIRS channels are marked (*p < 0.05).

## Discussion

In the present study, we examined the feasibility of implementing a compact multi-class hBCI with a minimal number of EEG channels and fNIRS SD pairs while maintaining BCI performance as high as possible. Our simulation study using data from 18 participants acquired as they performed three types of mental tasks demonstrated that an hBCI with only two EEG channels and two fNIRS SD pairs could yield a fairly high ternary classification accuracy of 77.6 ± 12.1% (theoretical chance level = 33.3%), which is thought to be high enough for practical BCI applications. In our previous study, in which we used the same dataset as the one used in this study, the ternary classification accuracy of the proposed hBCI was 82.2 ± 10.2% [20], which is approximately 5% higher than the accuracy attained by the system reported in the present study. Although the statistically significant loss of classification accuracy was observed (*p* < 0.05), the effect size was 0.42 which means that the effect is not large according to Cohen’s classification, and this difference does not seem to be very large considering that the previous results were achieved with a much higher number of sensors (21 EEG channels and six fNIRS SD pairs) than that used in this study (two EEG channels and two fNIRS SD pairs). Our results suggest that it would be feasible to implement a compact (or wearable) multi-class hBCI with fairly high performance for practical BCI applications.

Many previous studies have shown that the use of hBCIs can improve the classification accuracy of BCI systems; however, most of them used many EEG channels and fNIRS optodes. For example, Kaiser et al. [38] reported an average two-class classification accuracy of 76.7 ± 14.1% using six EEG channels and 33 fNIRS optodes, Fazli et al. [6] reported an average two-class classification accuracy of 83.2 ± 14.6% using 37 EEG channels and 24 fNIRS optodes, Shin et al. [10] used 30 EEG channels and 30 fNIRS optodes to achieve an average two-class classification accuracy of 83.6% (note that standard deviation was not provided), and Yin et al. [14] achieved an average classification accuracy of 89.0 ± 2.0% when discriminating between two different hand-clenching speed levels in motor imagery using 21 EEG channels and 18 fNIRS optodes. In a recent study, an hBCI system with reduced numbers of sensors, namely three EEG channels and 12 fNIRS optodes, was implemented [39]. This system exhibited an average binary classification accuracy of 81.2 ± 7.5% when discriminating between left- and right-hand MI tasks. However, in that study, the fNIRS signals were recorded at central areas over the primary motor cortex, in which case long and tedious preparatory work is generally inevitable for acquiring high-quality signals free from interference originating from hairs (especially dark dense hair) unless certain specialized equipment is employed [40]. Wang et al. [41] reported an average classification accuracy of 92.66 ± 1.11% in discriminating right-hand and foot MI tasks with only four EEG channels, which is the best MI classification performance ever achieved with such a small number of channels and is comparable to our accuracy of 91.1% achieved with five EEG channels. However, in Wang et al.’s study, optimal EEG channels were selected individually from larger number of channels. On the contrary, our results showed a high classification accuracy of 84.8 ± 13.4% with a common two-cahnnel EEG configuration (Cz and CP3). It is obvious that the individually optimized channels would yield better performance, but it should also be noted that the individualization approach requires an additional preparatory work to find the optimal channels from with a large number of channels and cannot be readily applied to wearable-type EEG devices.

We selected the number and placement of EEG sensors that maximized MI vs. IS classification accuracy and of fNIRS SD pairs that maximized MA vs. IS classification accuracy under the assumption that higher MI vs. IS EEG and MA vs. IS fNIRS classification accuracies would lead to higher three-class hybrid classification accuracy. To validate this assumption, we first calculated the spearman’s rank correlation coefficient (*r*) between the MI vs. IS EEG classification accuracies and three-class hBCI classification accuracies. All possible EEG channel pairs and the two optimal SD pairs (red dashed-line square in Fig 5(B)) were used to calculate the three-class hBCI classification accuracies. A significant positive correlation was found between the two types of classification accuracy (*r* = 0.944, 95% CI: 0.88–0.97, *p* < 0.001). As for the fNIRS aspect of the proposed system, we also evaluated the spearman’s rank correlation between the MA vs. IS and the three-class hBCI classification accuracies. All possible arrangements of two SD pairs (see S2 Table in S1 Text) and the (Cz, C3) EEG channel set were used to calculate the three-class hBCI classification accuracies. Similar to the EEG case, a significant positive correlation (*r* = 0.857, 95% CI: 0.15–0.98, *p* < 0.024) was found between the two types of classification accuracy.

An important issue that needs to be considered when implementing an hBCI system is selecting an appropriate number of mental tasks that can produce separable task-related brain signals. If a higher number of mental tasks that can produce distinguishable responses are employed, a higher information transfer rate can be achieved. For example, according to a previous study [26], breath holding (BH) task-related prefrontal fNIRS responses could be clearly discriminated from the MA- and IS-related ones. In addition, the fact that right-hand MI and left-hand MI induce discernible EEG signals has been well documented [34]. In this study, although only three mental tasks (MI, MA, and IS) were employed, the proposed hBCI could be expanded to a higher-order system (four or higher) by adopting a higher number of mental tasks, which is a promising research objective that we want to pursue in future studies.

The EEG and fNIRS data used in this study were recorded in a laboratory environment, and thus their practical usability should be further validated in the future studies. In addition, the participants recruited in this study were only healthy subjects; however, as people with severe neurological disorders might also have declined cognitive function. Therefore, it would be our future studies to implement a compact hBCI system combining the EEG and fNIRS systems based on the simulation results and evaluate the system with patients with severe disability in non-laboratory environments. In addition, the overall classification accuracy of the hBCI system also needs to be increased further for the system to be used in practical scenarios.

## Supporting information

S1 Text Supplementary information of detailed classification accuracies according to the EEG and fNIRS configurations and arrangement of the fNIRS optodes. Two tables and one figure are included in the Supplementary Information file: S1 Table. Two-channel EEG configurations and the corresponding MI vs. IS classification accuracies, S2 Table. NIRS SD arrangements and the corresponding MA vs. IS classification accuracies, and S1 Fig. Arrangement of the fNIRS optodes.
(PDF)
