# Supplementary material for: Toward a compact hybrid brain-computer interface (BCI): Performance evaluation of multi-class hybrid EEG-fNIRS BCIs with limited number of channels
Source: PLoS One. 2020 Mar 18;15(3):e0230491. doi: 10.1371/journal.pone.0230491 (PMC7080269; doi:10.1371/journal.pone.0230491)
Supplement: S1 Text — Two tables and one figure are included in the Supplementary Information file: S1 Table. Two-channel EEG configurations and the corresponding MI vs. IS classification accuracies, S2 Table. NIRS SD arrangements and the corresponding MA vs. IS classification accuracies, and S1 Fig. Arrangement of the fNIRS optodes. (PDF) [file pone.0230491.s001.pdf]

Toward a compact hybrid brain-computer interface (BCI): Performance evaluation of multi-class hybrid EEG-fNIRS BCIs with limited number of channels

Jinuk Kwon[1], Jaeyoung Shin[2], and Chang-Hwan Im[1*]

[1] Department of Biomedical Engineering, Hanyang University, Seoul, Korea
[2] Department of Electrical Engineering, Wonkwang University, Iksan, Korea

* Correspondence: ich@hanyang.ac.kr

Supplementary Information

Table 1 shows all the two-channel EEG configurations used to calculate the average MI vs. IS classification accuracies. The results are provided in descending order for classification accuracy. The four channel configurations that yielded the highest classification accuracies were used for Fig 4(b). Table 2 shows all the fNIRS SD arrangements used to calculate the average MA vs. IS classification accuracies for each number of SD pairs. The results are provided in descending order for classification accuracy for the same number of SD pairs. The location of the sources, detectors, and channels are shown in Figure 1. The fNIRS SD arrangements that yielded the highest classification accuracy for each number of SD pairs were used for Fig 5(b) in the main text.

Table 1. Two-channel EEG configurations and the corresponding MI vs. IS classification accuracies

Channel Configuration	Classification Accuracy (%)	Channel Configuration	Classification Accuracy (%)
(Cz, CP3)	84.8	(Cz, C5)	78.6
(Cz, C3)	84.1	(C5, C6)	77.9
(CP3, CP4)	83.3	(C4, FC4)	77.6
(C3, C4)	82.8	(CP4, FC4)	77.5
(C1, C2)	82.7	(C3, C5)	77.4
(CP3, FC3)	82.7	(C4, C2)	77.0
(C3, FC3)	82.5	(Cz, FC4)	76.9
(Cz, C1)	81.2	(C4, CP4)	76.6
(C3, C1)	81.0	(Cz, FC3)	75.4
(C3, CP3)	80.9	(Cz, C6)	75.3
(Cz, C4)	79.7	(C1, C5)	75.3
(FC3, FC4)	79.1	(C2, C6)	72.5
(Cz, CP4)	78.7	(C4, C6)	71.7
(Cz, C2)	78.6		

Table 2. NIRS SD arrangements and the corresponding MA vs. IS classification accuracies

Number of SD pairs	Sources	Detectors	Channels	Classification Accuracy (%)
6	all	all	all	82.6
5	2 to 6	2 to 6	all except 1, 6, 12	83.1
	1 to 5	1 to 5	all except 5, 11, 16	79.9
4	3 to 6	3 to 6	all except 1, 2, 6, 7, 12, 13	81.4
	2 to 5	2 to 5	all except 1, 5, 6, 11, 12, 16	79.7
	1 to 4	1 to 4	all except 4, 5, 10, 11, 15, 16	78.7
	1, 2, 5, 6	1, 2, 5, 6	all except 2, 3, 4, 8, 9, 13, 14, 15	75.4
3	4, 5, 6	4, 5, 6	4, 5, 9, 10, 11, 15, 16	80.1
	3, 4, 5	3, 4, 5	3, 4, 8, 9, 10, 14, 15	77.7
	1, 2, 3	1, 2, 3	1, 2, 6, 7, 8, 12, 13	74.8
	2, 3, 4	2, 3, 4	2, 3, 7, 8, 9, 13, 14	74.0
	1, 5, 6	1, 2, 6	1, 5, 6, 11	71.6
	1, 2, 6	1, 5, 6	6, 11, 12, 16	71.2
2	5, 6	5, 6	5, 10, 11, 16	74.4
	4, 5	4, 5	4, 9, 10, 15	74.2
	3, 4	3, 4	3, 8, 9, 14	72.8
	2, 3	2, 3	2, 7, 8, 13	72.7
	1, 2	1, 2	1, 6, 7, 12	71.7
	1, 6	1, 6	6, 11	67.8
	2, 5	2, 5	7, 10	67.5

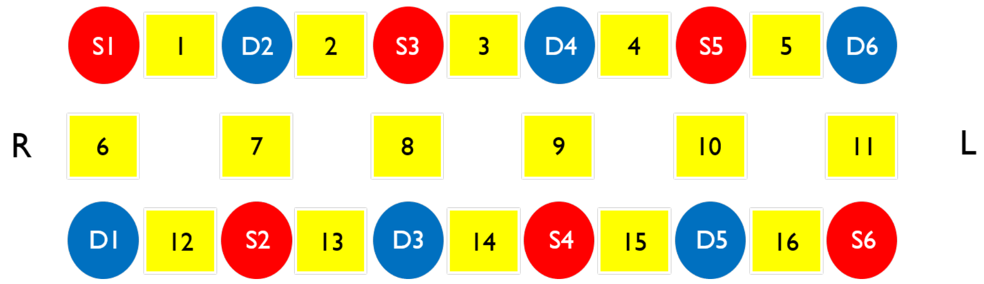

Figure 1. Arrangement of the fNIRS optodes (red: sources [S], green: detectors [D]) and channels (yellow)
